# Regional lymph node metastasis detected on preoperative CT and/or FDG-PET may predict early recurrence of pancreatic adenocarcinoma after curative resection

**DOI:** 10.1038/s41598-022-22126-y

**Published:** 2022-10-14

**Authors:** Ja Kyung Yoon, Mi-Suk Park, Seung-Seob Kim, Kyunghwa Han, Hee Seung Lee, Seungmin Bang, Ho Kyoung Hwang, Sang Hyun Hwang, Mijin Yun, Myeong-Jin Kim

**Affiliations:** 1 Department of Radiology and Research Institute of Radiological Science, Severance Hospital, Yonsei University College of Medicine, Seoul, Republic of Korea; 2 Department of Radiology and Research Institute of Radiological Science, Severance Hospital, Yonsei University College of Medicine, Seoul, Republic of Korea; 3 Division of Gastroenterology, Department of Internal Medicine, Yonsei University College of Medicine, Seoul, Republic of Korea; 4 Division of Hepatobiliary and Pancreas, Department of Surgery, Yonsei University College of Medicine, Seoul, Republic of Korea; 5 Department of Nuclear Medicine, Severance Hospital, Yonsei University College of Medicine, Seoul, Republic of Korea

**Keywords:** Cancer imaging, Pancreatic cancer

## Abstract

The objective of this study was to evaluate the role of regional lymph node (LN) metastasis detected on preoperative CT and/or ^18^F-fluoro-2-deoxyglucose-positron emission tomography (FDG-PET) scans in the prediction of early tumor recurrence after curative surgical resection of pancreatic ductal adenocarcinoma (PDAC). This retrospective study included 137 patients who underwent upfront surgery with R0 resection of PDAC between 2013 and 2016. Regional LN metastasis was identified using two criteria: positive findings for regional LN metastasis on either preoperative CT or FDG-PET scans (LN_OR_), or on both preoperative CT and FDG-PET scans (LN_AND_). A total of 55 patients had early tumor recurrence within 12 months after curative resection. Univariable and multivariable Cox proportional hazard regression analysis showed that preoperative carbohydrate antigen 19–9 (CA19-9) levels, preoperative locally advanced status, and regional LN metastasis (both LN_OR_ and LN_AND_ criteria) were significant risk factors for early recurrence. Positive LN_OR_ and LN_AND_ showed significantly poorer recurrence-free survival compared to negative regional LN metastasis groups (*p* = 0.048 and *p* = 0.020, respectively). Compared with the LN_AND_ criteria, the LN_OR_ criteria provided higher sensitivity (22.4% vs. 15.5%, *p* = 0.046) and a higher negative predictive value (61.9% vs. 59.8%, *p* = 0.046). The LN_OR_ definition provided more sensitive and accurate performance in diagnosing preoperative regional LN metastasis.

## Introduction

Pancreatic ductal adenocarcinoma (PDAC) is notorious for its poor prognosis. Although curative resection is desirable, many patients are diagnosed in an unresectable state^1,2^. Even in resectable or borderline resectable cases according to the National Comprehensive Cancer Network (NCCN) guidelines, the tumor recurrence rate is also high^3–5^. In addition, while R0 resection is one of the most important factors that influence prognosis, recurrences occur even in these cases^6,7^.

Regional LN metastasis have been reported in 14%–75% of patients with resectable PDAC^8–10^. Although regional LN metastasis is not a determining factor in assessing resectability per the NCCN guidelines, the NCCN guideline recommends considering neoadjuvant therapy in the presence of large regional LNs on preoperative imaging studies^11^. Regional LN metastasis indicates a high risk of disseminated disease and has been identified as a risk factor for early recurrence after surgical resection^11–14^. However, the impact of preoperatively diagnosed regional LN metastasis on early recurrence has been seldom reported with conflicting results^15–17^. As such, the clinical implications of regional LN metastasis identified on preoperative computed tomography (CT) or ^18^F-fluoro-2-deoxyglucose-positron emission tomography (FDG-PET) scans remain controversial^2,4,18^.

Indeed, conventional imaging modalities, including CT and FDG-PET scans, are often insufficient for the preoperative diagnosis of LN metastasis in PDAC patients^19^. CT studies have low sensitivity (7%–37%) with a more acceptable specificity (64–99%) in determining regional LN metastasis^2,20–22^. In addition, there are no established criteria for determining metastatic LNs on CT scans^2^. The advantages of FDG-PET scans include the capability to detect micro-metastases in small or normal-sized regional LNs, with slightly better performance (sensitivity: 30%–49%, specificity: 63%–93%) compared with CT scans^20,23,24^. However, evidence showing the performance of imaging studies in identifying regional LNs preoperatively are limited. Hence, collective or combined regional LN metastasis identification using CT and/or FDG-PET scans may help improve the diagnostic performance.

Therefore, this study aimed to evaluate the role of regional LN metastasis identified on preoperative CT and/or FDG-PET scans in predicting early tumor recurrence after curative surgical resection of PDAC.

## Results

The baseline characteristics of the included patients are shown in Table 1. The median preoperative carbohydrate antigen 19–9 (CA19-9) level was 68.9 U/mL. Positive FDG uptake of the tumor was noted in 82.5% (113/137) of cases, with significant correlation with tumor size (Supplementary Table 1). Of all the tumors detected, 49.6% (68/137) were in the pancreatic head, and 82.5% (113/137) were classified as resectable according to preoperative imaging scans. PPPD (pylorus-preserving pancreaticoduodenectomy) was performed in 48.2% (66/137) of the patients. Regional LN metastasis was suggested in 13.9% (19/137) and 10.9% (15/137) of the patients according to the LN_OR_ and LN_AND_ definitions, respectively. Pathologic regional LN metastasis was confirmed in 42.3% (58/137) of the patients. Adjuvant therapy was administered in 73.7% (101/137) of the patients who received chemotherapy and/or concurrent chemoradiation therapy. Death occurred in 37.2% (51/137) of the patients, and recurrence was detected in 69.3% (95/137) of the patients. Among the 95 patients who developed recurrence, 57.9% (55/95) had early recurrence. The median overall survival (OS) was 34.1 months (interquartile range (IQR), 15.0–51.2 months), while the median recurrence-free survival (RFS) was 15.4 months (IQR, 6.6–43.4 months).

**Table 1:** Baseline characteristics of the study population.

Variables	Values
Age*	64.9 ± 10.0
Male	79 (57.7%)
BMI (kg/m^2^)*	23.0 ± 2.8
Preoperative CA19-9 (U/mL)^†^	68.9 (19.6–284.8)
Head location	68 (49.6%)
Tumor size^†^	25.0 (18.0–31.0)
Positive uptake of tumor on FDG-PET scan	113 (82.5%)
LN_OR_	19 (13.9%)
LN_AND_	15 (10.9%)
**NCCN resectability**	
Resectable	113 (82.5%)
Borderline resectable	14 (10.2%)
Locally advanced	10 (7.3%)
**Type of surgery**	
PPPD	66 (48.2%)
Distal pancreatectomy	63 (46.0%)
Total pancreatectomy	8 (5.8%)
**Pathologic T stage (pT)**^**‡**^	
T1	10 (7.3%)
T2	27 (19.8%)
T3	99 (72.3%)
T4	1 (0.7%)
Pathologic LN metastasis (pN) ^‡^	58 (42.3%)
Adjuvant therapy	101 (73.7%)
**Recurrence**	95 (69.3%)
Early recurrence	55 (57.9%)
**Location of recurrence**	
Locoregional recurrence	31 (22.6%)
Distant recurrence	64 (46.7%)
Death	51 (37.2%)
Overall survival (months)^†^	34.1 (15.0–51.2)
Recurrence-free survival (months)^†^	15.4 (6.6–43.4)

Data are presented as numbers (%) unless otherwise indicated. *Data are presented as mean ± standard deviation. ^†^Data are presented as median (1st quartile–3rd quartile). ^‡^TNM staging according to the 8th edition of the American Joint Committee on Cancer (AJCC)/Union for International Cancer Control (UICC) TNM staging system. *BMI*, body mass index; *CA19-9*, carbohydrate antigen 19-9; *FDG-PET*, ^18^F-fluoro-2-deoxyglucose-positron emission tomography; *LN*_*OR*_, regional LN metastasis on preoperative CT or FDG-PET scans; *LN*_*AND*_, regional LN metastasis on preoperative CT and FDG-PET scans; *NCCN*, National Comprehensive Cancer Network; *PPPD*, pylorus-preserving pancreaticoduodenectomy.

### Preoperative characteristics according to recurrence group

**Figure 1:**
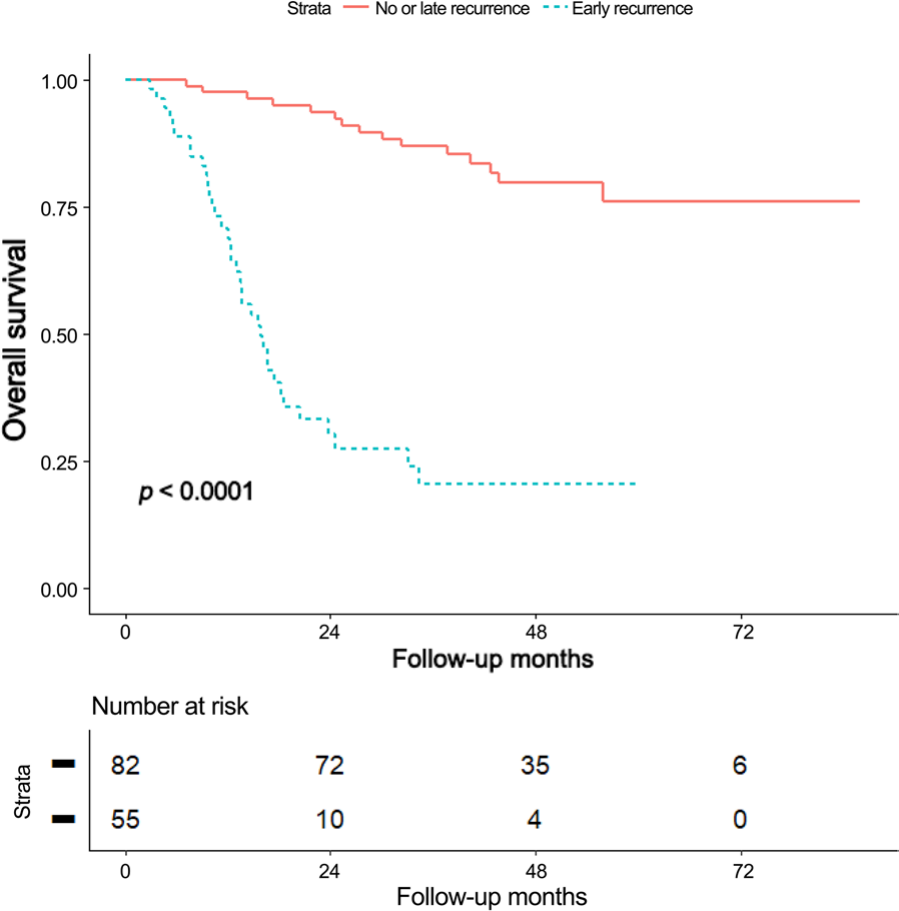
Overall survival (OS) of pancreatic ductal adenocarcinoma (PDAC) patients according to the recurrence group. The Kaplan-Meier curve showed that the early recurrence group had significantly poorer OS than the no or late recurrence groups.

The differences in preoperative risk factors between the early recurrence group and the no or late recurrence group are shown in Table 2. Significant differences between the two recurrence groups were observed in the NCCN resectability (*p* = 0.040) and regional LN metastasis by both LN_OR_ and LN_AND_ definitions (*p* = 0.014 and *p* = 0.012, respectively). There were significantly more deaths in the early recurrence group (65.5% (36/55) vs. 18.3% (15/82)) compared to the no or late recurrence group. The early recurrence group showed shorter OS than the no or late recurrence group (median 13.6 months vs. 43.8 months) (Fig. 1). On comparison of early recurrence and late recurrence groups, LN_OR_ occurred more frequently in the early recurrence group than in the late recurrence group (23.6% (13/55) vs. 5.0% (2/40), *p* = 0.030) (Supplementary Table 2. Death also occurred more frequently in the early recurrence group than in the late recurrence group (65.5% (36/55) vs. 27.5% (11/40), *p* = 0.001), with significantly shorter OS (median 13.6 months vs. 38.4 months, *p* < 0.001).

**Table 2:** Comparison of preoperative characteristics according to the status of tumor recurrence.

Variables	No or late recurrence (n = 82)	Early recurrence (n = 55)	*p*-value
Age^*^	65.4 ± 9.3	64.0 ± 11.1	0.445
Male	36 (43.9%)	22 (40.0%)	0.782
BMI (kg/m^2^)*	23.2 ± 2.7	22.8 ± 2.9	0.420
Preoperative CA19-9 (U/mL)^†^	56.5 (16.6–232.5)	108.2 (22.8–329.6)	0.238
Head location	36 (43.9%)	32 (58.2%)	0.143
Tumor size^†^	24.5 (17.0–32.0)	25.0 (20.0–30.0)	0.871
Positive uptake of tumor on FDG-PET scan	65 (79.3%)	48 (87.3%)	0.362
LN_OR_	6 (7.3%)	13 (23.6%)	**0.014**
LN_AND_	4 (4.9%)	11 (20.0%)	**0.012**
NCCN resectability			**0.040**
Resectable	73 (89.0%)	40 (72.7%)	
Borderline resectable	6 (7.3%)	8 (14.5%)	
Locally advanced	3 (3.7%)	7 (12.7%)	
Death	15 (18.3%)	36 (65.5%)	**< 0.001**
Overall survival (months)^†^	43.8 (32.3–57.2)	13.6 (9.6–21.4)	< **0.001**

Data are presented as numbers (%) with the *p*-values of Fisher’s exact test unless otherwise indicated. *Data are presented as mean ± standard deviation, with the *p*-value of Student’s t-test. ^†^Data are presented as median (1st quartile–3rd quartile), with the *p*-value of the Mann–Whitney U test. *BMI*, body mass index; *CA19-9*, carbohydrate antigen 19–9; *FDG-PET*, ^18^F-fluoro-2-deoxyglucose-positron emission tomography; *LN*_*OR*_, regional LN metastasis on preoperative CT or FDG-PET scans; *LN*_*AND*_, regional LN metastasis on preoperative CT and FDG-PET scans; *NCCN*, National Comprehensive Cancer Network.

Significant values are in [bold].

### Preoperative risk factors for early recurrence

**Figure 2:**
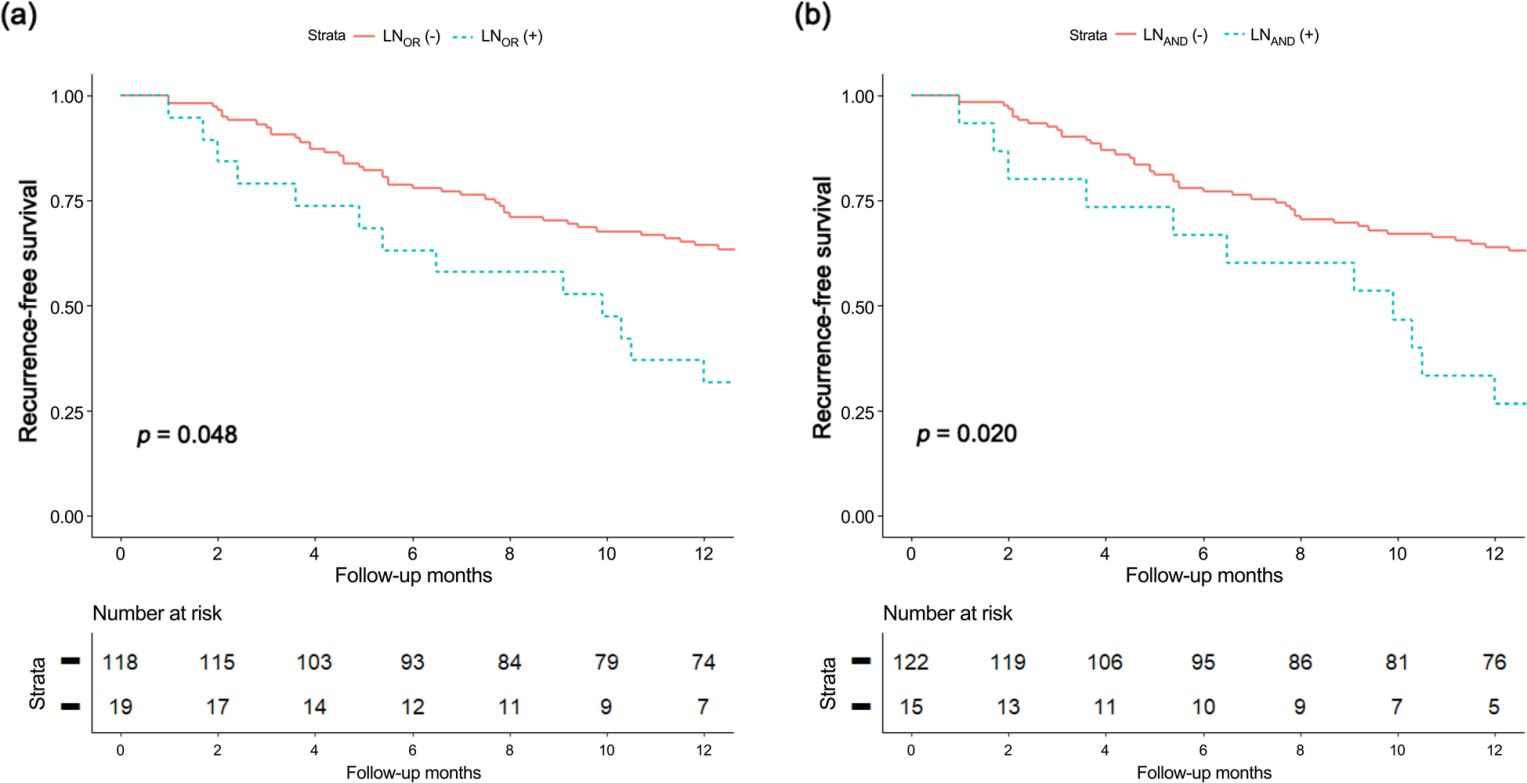
Recurrence-free survival (RFS) according to the status of regional lymph node (LN) metastasis on preoperative images. The positive regional LN metastasis group according to both (**a**) LN_OR_ and (**b**) LN_AND_ criteria showed significantly lower RFS than the no regional LN metastasis group.

**Figure 3:**
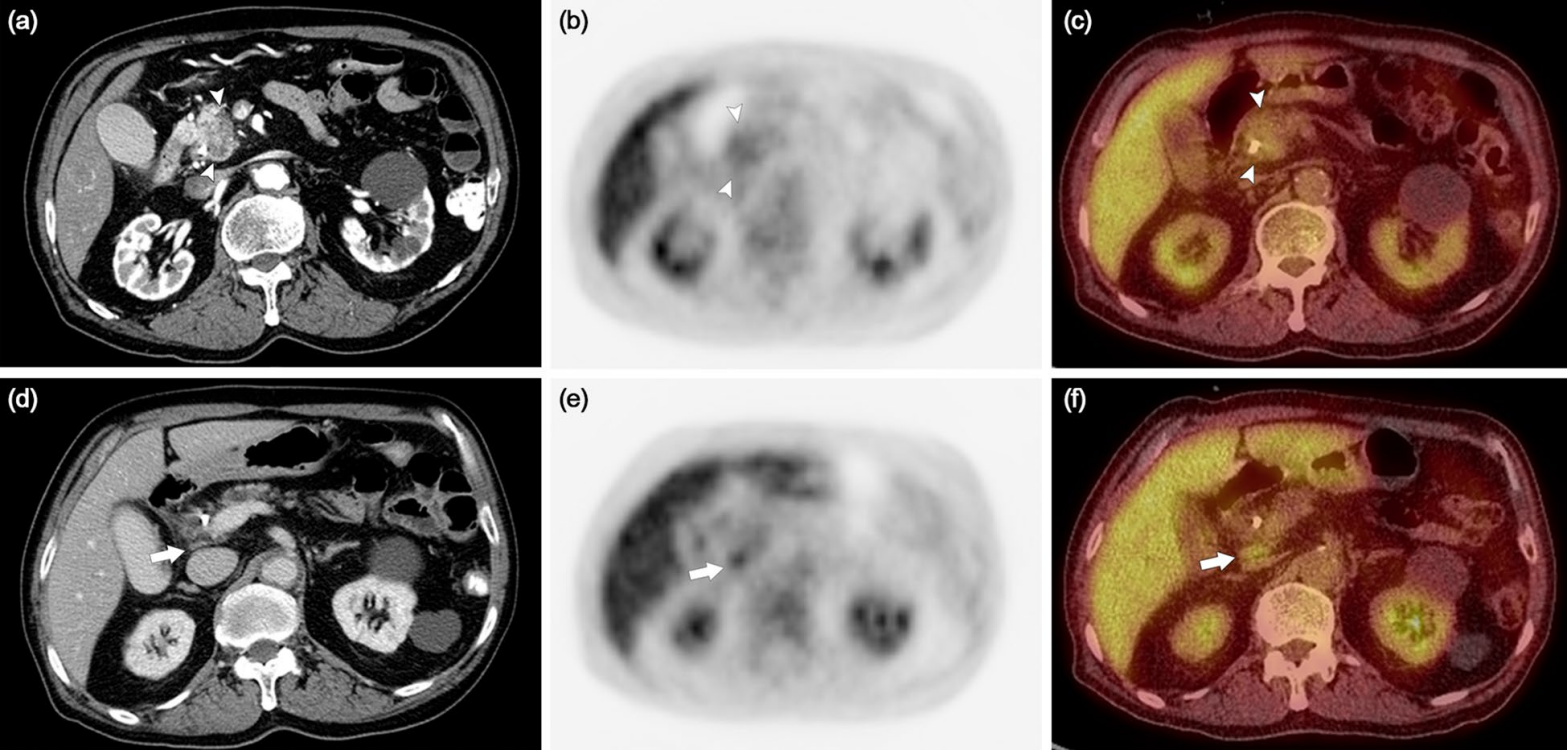
Contrast-enhanced dynamic computed tomography (CT) and ^18^F-fluoro-2-deoxyglucose-positron emission tomography (FDG-PET) scans of a 76-year old male patient diagnosed with pancreatic head cancer. A 2.7 cm sized hypoattenuating mass (arrowheads) is noted on the arterial phase of the CT (**a**), which shows significant FDG uptake on the FDG-PET (**b**) and FDG-PET/CT scans (**c**). A small lymph node (LN) was noted in the portocaval space (arrow) on the venous phase of the CT (**d**), with significant FDG uptake on the FDG-PET (**e**) and FDG-PET/CT scans (**f**). The patient was categorized as positive regional LN metastasis by the LN_OR_ definition, but negative regional LN metastasis by the LN_AND_ definition. After pylorus-preserving pancreaticoduodenectomy and regional LN dissection, regional LN metastasis was confirmed. Early local recurrence was identified on CT and FDG-PET scans performed 10.5 months after surgery. Patient expired 15.9 months after surgery.

On univariable Cox proportional hazard analysis of early recurrence, preoperative CA19-9 level (× 100 U/mL), preoperative locally advanced status, LN_OR_, and LN_AND_ were significant risk factors. On multivariable Cox proportional hazard analysis using either LN_OR_ or LN_AND_ definitions, regional LN metastasis by any definition was a significant risk factor for early recurrence, along with preoperative CA19-9 levels and preoperative locally advanced status (Table 3). Positive regional LN metastasis according to the LN_OR_ (Fig. 2a) and LN_AND_ (Fig. 2b) definitions showed significantly poorer RFS (*p* = 0.048 and *p* = 0.020, respectively). A representative case is shown in Fig. 3.

**Table 3:** Univariable and multivariable Cox proportional hazard analyses between preoperative characteristics and early recurrence.

Variables	Univariable			Multivariable with LN_OR_			Multivariable with LN_AND_		
	HR	95% CI	*p*-value	HR	95% CI	*p*-value	HR	95% CI	*p*-value
Age	0.989	0.963–1.016	0.417	–	–	–	–	–	–
Male sex	1.180	0.688–2.025	0.547	–	–	–	–	–	–
BMI (kg/m^2^)	0.953	0.864–1.051	0.338	–	–	–	–	–	–
Preoperative CA19-9 (× 100 U/mL)	1.008	1.002–1.015	**0.012**	1.009	1.002–1.016	**0.010**	1.011	1.004–1.017	**0.003**
Tumor size	0.996	0.981–1.011	0.563	–	–	–	–	–	–
Head location	1.523	0.891–2.604	0.124	–	–	–	–	–	–
Positive uptake of tumor on FDG-PET scan	1.385	0.543 – 3.529	0.495						
NCCN resectability			**0.020**			** < 0.001**			**0.023**
Resectable	1	Reference		1	Reference		1	Reference	
Borderline resectable	1.748	0.817–3.737	0.150	1.551	0.705–3.413	0.275	1.382	0.732–2.612	0.319
Locally advanced (unresectable)	2.797	1.249–6.262	**0.012**	2.445	1.073–5.571	**0.033**	2.649	1.275–5.507	**0.009**
LN_OR_	2.382	1.278–4.442	**0.006**	2.027	1.057–3.885	**0.033**	–	–	–
LN_AND_	2.494	1.287–4.835	**0.007**	–	–	–	2.086	1.054–4.125	**0.035**

*HR,* hazard ratio; *CI,* confidence interval; *BMI*, body mass index; *CA19-9*, carbohydrate antigen 19–9; *FDG-PET*, ^18^F-fluoro-2-deoxyglucose-positron emission tomography; *LN*_*OR*_, regional LN metastasis on preoperative CT or FDG-PET scans; *LN*_*AND*_, regional LN metastasis on preoperative CT and FDG-PET scans; *NCCN*, National Comprehensive Cancer Network.

Significant values are in [bold].

### *Regional LN metastasis by LN*_*OR*_* and LN*_*AND*_* definitions*

Regional LN metastasis according to the LN_OR_ and LN_AND_ definitions was compared to the pathologic regional LN metastasis as the reference standard (Table 4). Compared with the LN_AND_ criteria, the LN_OR_ definition provided a significantly higher sensitivity (22.4% vs. 15.5%, *p* = 0.046) and negative predictive value (NPV; 61.9% vs. 59.8%, *p* = 0.046). The LN_OR_ definition also provided a higher positive predictive value (PPV; 68.4% vs. 60.0%, *p* = 0.122) and accuracy (62.8% vs. 59.9%, *p* = 0.134), but without statistical significance. No difference was observed in the specificity between the two definitions (92.4%).

**Table 4:** Diagnostic performance of regional LN metastasis by LN_OR_ and LN_AND_ definitions.

	Sensitivity	Specificity	PPV	NPV	Accuracy (95% confidence interval)	
LN_OR_	0.224	0.924	0.684	0.619	0.628 (0.541–0.709)	
LN_AND_	0.155	0.924	0.600	0.598	0.599 (0.511–0.681)	
*p*-value	0.046*	1.000*	0.122^†^	0.046^†^	0.134*	

*PPV,* positive predictive value; *NPV,* negative predictive value; *LN*_*OR*_, regional LN metastasis on preoperative CT or FDG-PET (^18^F-fluoro-2-deoxyglucose-positron emission tomography) scans; *LN*_*AND*_, regional LN metastasis on preoperative CT and FDG-PET scans; *McNemar’s test was used. ^†^Generalised estimating equation was used.

## Discussion

This study showed that preoperative CA19-9 level, NCCN resectability, and regional LN metastasis can be used to identify patients at risk of developing early tumor recurrence after undergoing surgical resection. Regional LN metastasis according to the LN_OR_ and LN_AND_ definitions was a significant risk factor for early recurrence, with the LN_OR_ definition showing higher sensitivity than the LN_AND_ definition.

PDAC patients have a high incidence of regional LN metastasis, with a positivity rate of up to 80% in those with resectable PDAC^25^. In this study, regional LN metastasis detected on preoperative imaging was a significant risk factor for early tumor recurrence, which is consistent with previous reports^13,15^. However, the preoperative identification of regional LN metastasis on imaging studies remains challenging due to the lack of consensus on the diagnostic criteria and overall low sensitivity^20,21,23,26^. Our results showed that both the LN_OR_ and LN_AND_ definitions provided sensitivity, specificity, PPV, NPV, and accuracy which fell within the range of previous reports^2,27–30^. The LN_OR_ definition provided more sensitive and accurate performance in predicting regional LN metastasis by identifying LNs with any signs of positive metastasis on either preoperative CT or FDG-PET scans. Considering that the aim of preoperative imaging studies may be in discerning those patients who will benefit from curative surgery, the more sensitive LN_OR_ definition may be more appropriate in the preoperative setting to help guide the course of treatment. In addition, performing both CT and FDG-PET may be necessary in correctly identifying regional LN metastasis with high diagnostic accuracy, helping to better predict the risk of early tumor recurrence after curative resection. Further efforts to improve the diagnostic performance for preoperative regional LN metastasis are necessary.

Despite its relatively low diagnostic performance, the preoperative diagnosis of LN metastasis based on CT or FDG-PET is a significant risk factor for early recurrence. Significant differences were observed in the RFS according to the presence of regional LN metastasis. Moreover, PDAC with high-risk factors but without distant metastasis at initial diagnosis is still considered as a systemic disease, even in resectable cases, suggesting the necessity of neoadjuvant therapy in patients at an increased risk of early tumor recurrence^11,31^. Our results suggest that positive regional LN metastasis on preoperative CT or FDG-PET could serve as a practical guide for determining the appropriate treatment.

The preoperative CA19-9 level was a significant factor for early tumor recurrence in our study. Previous studies have also identified CA19-9 level as a useful predictor of early tumor recurrence within 6 months or 12 months after curative resection, poor OS and RFS^4,32–34^. However, although any elevation in the CA19-9 level is considered as a poor prognostic factor, there is no consensus on the cutoff of CA19-9 level^4,33–38^. A dichotomous analysis of the CA19-9 cut-off values for a higher risk of early recurrence has yielded a wide range of values, from 50 U/mL to 529 U/mL^4,32–34^. Others showed that the analysis of CA19-9 level as continuous variable better reflects the proportional risk of early recurrence, suggesting that dichotomisation of CA19-9 level diminishes its predictive power^34,39^. In this study, we used the preoperative CA19-9 value divided by 100 which may provide easier calculation than the previously used log values, thereby facilitating the prediction of early tumor recurrence in the clinical setting^34,39^.

NCCN resectability is a widely accepted and utilised prognostic risk factor for tumor recurrence and OS^13,39^. In our study, even the locally advanced group successfully underwent R0 resection, but showed significantly lower RFS compared to the resectable group. Therefore, these patients may be at higher risk for early tumor recurrence despite undergoing with R0 resection and may benefit from other treatment methods^40,41^. On the contrary, the RFS of the borderline resectable group was not significantly different from that of both resectable and locally advanced groups, which may indicate the heterogeneity of the borderline resectable group in terms of early tumor recurrence. Therefore, the borderline resectable group may be better classified using additional factors in addition to the tumor-vessel relationship, such as regional LN status and CA19-9 level as suggested by the recent consensus guidelines^42^.

Our study has several limitations. First, its retrospective nature included inherent bias. Second, patients with Lewis-negative pancreatic cancer was not accounted for in this study. Third, preoperatively suspected regional LN metastasis was not pathologically confirmed in a node-by-node manner, leading to difficulties in assessing the diagnostic accuracy of LN metastasis identified on preoperative scans. In addition, N1 or N2 was not divided based on the number of positive LNs, as suggested by the 8th American Joint Committee on Cancer (AJCC)/Union for International Cancer Control (UICC) TNM staging system.

In conclusion, preoperative LN metastasis detected on CT or FDG-PET scans, along with elevated serum CA19-9 levels, and locally advanced status were significant risk factors for predicting early recurrence of PDAC after surgery. The LN_OR_ definition provided more sensitive and accurate performance in diagnosing preoperative regional LN metastasis.

## Methods

This retrospective study was approved by the Institutional Review Board at Yonsei University College of Medicine, and was conducted in accordance with the Declaration of Helsinki as revised in 2013. Informed consent was obtained from all patients enrolled in this study. The requirement for written informed consent was waived owing to the retrospective nature of the study.

### Study population

**Figure 4:**
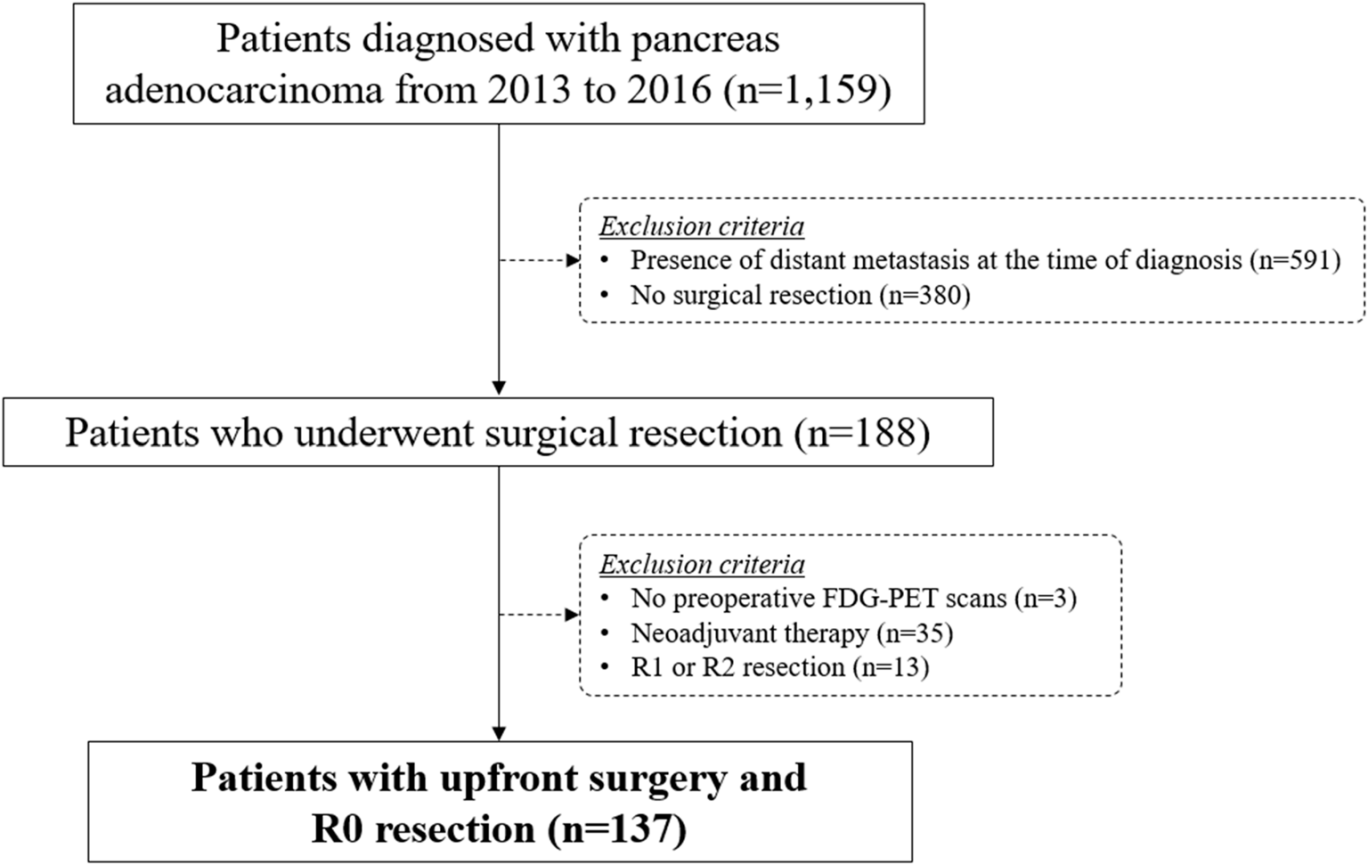
Flow diagram of patients included in this study. From an existing cohort of patients with pathologically proven pancreatic adenocarcinoma from 2013 to 2016, a total of 137 patients who underwent upfront surgery and successful R0 resection were eligible for the final analysis. *FDG-PET*, ^18^F-fluoro-2-deoxyglucose-positron emission tomography.

The institutional cohort of the PDAC registry from a tertiary hospital (Yonsei University Medical Center, Seoul, Korea) was retrospectively searched to collect consecutive data of patients with pathologically confirmed PDAC between January 2013 and December 2016 (Fig. 4). A total of 1,159 patients were identified. Patients diagnosed with PDAC who underwent curative surgical resection at our institution were included in the study. Exclusion criteria included 1) disseminated disease at diagnosis, 2) no surgical resection, 3) R1 or R2 resection, 4) administration of neoadjuvant therapy (either chemotherapy or concurrent chemoradiation therapy), and 5) no preoperative FDG-PET scans. A total of 137 patients who underwent upfront surgery resulting in R0 resection were included in the final analysis.

### Medical record and image review

The PDAC cohort was compiled by two radiologists after independently reviewing the preoperative CT and FDG-PET scans, which were carried out as routine preoperative work-up at our institution. The two readers were blinded to the clinical and pathologic information of the patients. From this cohort, results of the CT and FDG-PET scans performed within one month prior to surgery, age, sex, and serum CA19-9 level of the eligible patients were obtained. The tumor location was categorised as head (including head, uncinate process, and neck) or body/tail. The tumor size was categorised as 20 mm and smaller in size, or larger than 20mm adapted from the 8th AJCC/UICC TNM staging system^43^. The presence or absence of the main pancreatic duct or bile duct dilatation were recorded. Tumor resectability was evaluated based on the NCCN guidelines (version 2.2021)^11^. Clinical T stage was classified according to the 8th edition of the AJCC/UICC TNM staging system^43^. The regional LNs for PDAC in the pancreatic head were found in the supra- or infra-pyloric areas, along the common hepatic artery, along the hepatoduodenal ligament, around the celiac artery, retropancreatic area, and peripancreatic area^44^. The regional LNs for PDAC at body or tail location included LNs along the common hepatic artery, hepatoduodenal ligament, splenic hilum, and retropancreatic, peripancreatic areas, and the mesenteric root areas^44^. Regional LN metastasis on contrast-enhanced CT was identified when LNs showed increased size (short axis larger than 10 mm) or necrosis^45^. Positive FDG uptake was defined as increased FDG accumulation compared with the surrounding tissues that was not related to normal physiologic uptake. LN_OR_ was defined as a regional LN metastasis identified on contrast-enhanced CT **or** FDG-PET scans, while LN_AND_ was defined as a regional LN metastasis identified on contrast-enhanced CT findings **and** FDG-PET scans^20,46^. Pancreatic CT was performed as recommended by NCCN guidelines, and detailed protocol of FDG-PET scan are summarized (Supplementary Methods 1 and 2)^11,47^.

### Postoperative follow-up and diagnosis of tumor recurrence

Postoperative surveillance included CA19-9 measurement, contrast-enhanced abdomen-pelvic CT, FDG-PET, and chest CT scans. Tumor recurrence was diagnosed using imaging studies. In patients where imaging findings were inconclusive, tumor recurrence was confirmed by pathologic diagnosis. Early recurrence was defined as tumor recurrence within 12 months after surgery^14^. OS was defined as the period from the date of diagnosis to the date of death or last follow-up. RFS was defined as the period from the date of surgery to the date of tumor recurrence or last follow-up. Patients were routinely followed up using CT, serum CA19-9 and CEA levels at intervals of two or three months until at least 12 months after surgery. Post-operative adjuvant therapy (chemotherapy with or without radiation therapy) was administered at the clinician’s discretion, according to the pathologic findings.

### Statistical analysis

All analyses were performed using a commercial software (R version 4.2.1, R Foundation for Statistical Computing, Vienna, Austria)^48^. Continuous variables between the recurrence groups were compared using Student’s t-test for parametric values and Mann-Whitney U-test for non-parametric variables. The Fisher’s exact test was used to compare the frequencies of variables between the two recurrence groups. Spearman’s correlation was used to analyse the correlation between tumor size, positive FDG uptake of the tumor, and positive FDG uptake of regional LNs. Kaplan-Meier survival analyses with log-rank tests were used to determine the OS and RFS. Cox proportional hazard regression analysis was used to determine the preoperative risk factors for early tumor recurrence. Considering the wide range of CA19-9 values (range, 0.1–20,000 U/mL), the preoperative CA19-9 level divided by 100 (× 100 U/mL) was utilized to facilitate data manipulation. The diagnostic performances of LN_OR_ and LN_AND_ definitions were determined based on the pathologically confirmed metastatic regional LNs as a reference standard on a per-patient basis using confusion matrix analyses. The performances of LN_OR_ and LN_AND_ were compared using the McNemar’s test to determine the sensitivity and specificity, and the generalised estimating equation for PPV and NPV. A *p*-value of < 0.05 was considered significant.

## Supplementary Information

Supplementary Information.

## Data Availability

The datasets generated or analysed during the study are available from the corresponding author on reasonable request.
