# Supplementary material for: Regional lymph node metastasis detected on preoperative CT and/or FDG-PET may predict early recurrence of pancreatic adenocarcinoma after curative resection
Source: Sci Rep. 2022 Oct 14;12:17296. doi: 10.1038/s41598-022-22126-y (PMC9568602; doi:10.1038/s41598-022-22126-y)
Supplement: Supplementary file 1 — Supplementary Information. [file 41598_2022_22126_MOESM1_ESM.docx]

**Supplementary Table 1. Correlation between positive uptake of the tumor and regional LNs on FDG-PET scan and tumor size.**

|  | **Spearman's rho** | ***p*-value** |
| --- | --- | --- |
| **Tumor size – positive uptake of the tumor on FDG-PET scan** | 0.176 | **0.040** |
| **Tumor size – positive uptake of regional LNs on FDG-PET scan** | -0.270 | 0.264 |
| **Positive uptake of the tumor on FDG-PET scan  – positive uptake of regional LNs on FDG-PET scan** | 1.000 | **<0.001** |

*FDG-PET*, ^18^F-fluoro-2-deoxyglucose-positron emission tomography.

**Supplementary Table 2. Comparison of preoperative characteristics of the early recurrence and late recurrence groups**

| **Variables** | |  | **Late recurrence  (n=40)** | | |  | **Early recurrence  (n=55)** | | |  | ***p*-value** |
| --- | --- | --- | --- | --- | --- | --- | --- | --- | --- | --- | --- |
| Age^*^ | |  | 65.4 ± 9.5 | | |  | 64.0 ± 11.1 | | |  | 0.537 |
| Male sex | |  | 20 | (50.0%) | |  | 22 | (40.0%) | |  | 0.447 |
| BMI (kg/m^2^)^*^ | |  | 23.7 ± 2.7 | | |  | 22.8 ± 2.9 | | |  | 0.136 |
| Preoperative CA19-9 (U/mL)^†^ | |  | 104.3 (21.6–317.9) | | |  | 108.2 (22.8–329.6) | | |  | 0.821 |
| Positive uptake of tumor on FDG-PET scan | |  | 38 | (95.0%) | |  | 48 | (87.3%) | |  | >0.999 |
| LN_OR_ | |  | 2 | (5.0%) | |  | 13 | (23.6%) | |  | **0.030** |
| LN_AND_ | |  | 2 | (5.0%) | |  | 11 | (20.0%) | |  | 0.072 |
| NCCN resectability | |  |  |  |  |  |  |  |  |  | 0.332 |
|  | Resectable |  | 34 | (85.0%) | |  | 40 | (72.7%) | |  |  |
|  | Borderline resectable |  | 8 | (14.5%) | |  | 8 | (14.5%) | |  |  |
|  | Locally advanced |  | 7 | (12.7%) | |  | 7 | (12.7%) | |  |  |
| Location of recurrence | |  |  |  |  |  |  |  |  |  | 0.521 |
|  | Locoregional recurrence |  | 15 | (37.5%) | |  | 16 | (29.1%) | |  |  |
|  | Systemic recurrence |  | 25 | (62.5%) | |  | 39 | (70.9%) | |  |  |
| Death | |  | 11 | (27.5%) | |  | 36 | (65.5%) | |  | **0.001** |
| Overall survival (months)^†^ | |  | 38.4 (29.6–47.9) | | |  | 13.6 (9.6–21.4) | | |  | **<0.001**^†^ |

Data are presented as numbers (%), with the *p*-values of Fisher’s exact test unless otherwise indicated. ^*^Data are presented as mean ± standard deviation, with the *p*-value of Student’s t-test. ^†^Data are presented as median (1^st^ quartile–3^rd^ quartile), with the *p*-value of the Mann-Whitney U test. *BMI*, body mass index; *CA19-9*, carbohydrate antigen 19-9; *FDG-PET*, ^18^F-fluoro-2-deoxyglucose-positron emission tomography; *LN_OR_*, regional LN metastasis on preoperative CT or FDG-PET scans; *LN_AND_*, regional LN metastasis on preoperative CT and FDG-PET scans; *NCCN*, National Comprehensive Cancer Network.

**Supplementary Methods 1. Detailed Protocol of Pancreatic CT**

All patients underwent CT examinations with a pancreatic protocol using a 64-channel scanner (Sensation 64, Siemens; Brilliance 64; Philips Healthcare). After obtaining noncontrast CT images, contrast-enhanced pancreatic CT scanning was performed with intravenous administration of nonionic contrast media (Iopamiro 370, Bracco [iopamidol], 1.62 mL/kg). Pancreatic and portal venous phase images were obtained by adding 23 seconds to the time of peak abdominal aortic enhancement calculated at the hepatic hilum and 25 seconds to the end of the pancreatic phase. The scanning parameters were as follows: beam collimation, 0.6 mm; slice thickness, 3 mm; reconstruction interval, 3 mm; rotation time, 0.5 seconds; effective tube current-time charge, 150–250 mAs; 100 kVp.

**Supplementary Methods 2. Detailed Protocol of FDG-PET scan**

All ^18^FDG PET/PET-CT scans were performed with a dedicated PET/CT scanner (Discovery STe, GE Healthcare; or Biograph TruePoint 40, Siemens Healthcare). All patients fasted for at least 6 h prior to the PET/CT scan. A dose of approximately 5.5 MBq/kg of 18F-FDG was intravenously injected 60 min before imaging. First, CT scans were performed at 30 mA and 130 kVp with the Discovery STe scanner or at 36 mA and 120 kVp with the Biograph TruePoint scanner without contrast-enhancement. After the CT scan was complete, a PET scan was performed from the neck to the proximal thigh, with an acquisition time of 3 min per bed position in a 3D mode. PET images were reconstructed using ordered subset expectation maximization with attenuation correction
